# Supplementary figures and images for: AARS2 ameliorates myocardial ischemia via fine-tuning PKM2-mediated metabolism
Source: eLife. 2025 May 15;13:RP99670. doi: 10.7554/eLife.99670 (PMC12080999; doi:10.7554/eLife.99670)

Figure 1A

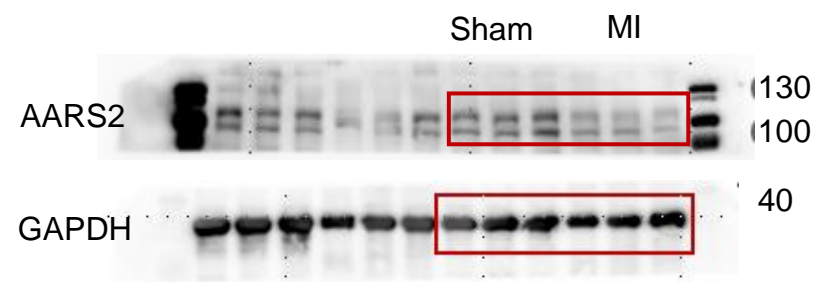

Figure 1D

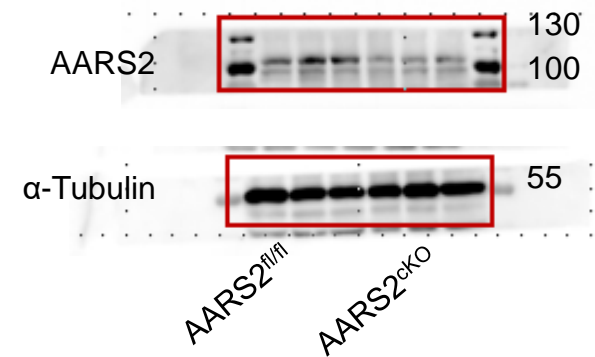

Supplement: Figure 1—source data 1. [file elife-99670-fig1-data1.zip › Figure 1-source data 1. PDF file containing original western blots for Figure 1A and 1D.pdf]

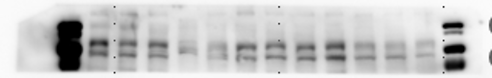

Supplement: Figure 1—source data 2. [file elife-99670-fig1-data2.zip › -Figure 1-source data 2. Original files for western blot analysis displayed in Figure 1A and 1D/Figure 1A/AARS2.tif]

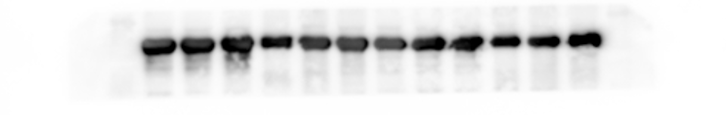

Supplement: Figure 1—source data 2. [file elife-99670-fig1-data2.zip › -Figure 1-source data 2. Original files for western blot analysis displayed in Figure 1A and 1D/Figure 1A/GAPDH.tif]

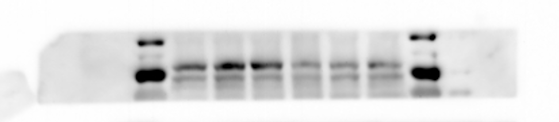

Supplement: Figure 1—source data 2. [file elife-99670-fig1-data2.zip › -Figure 1-source data 2. Original files for western blot analysis displayed in Figure 1A and 1D/Figure 1D/AARS2.tif]

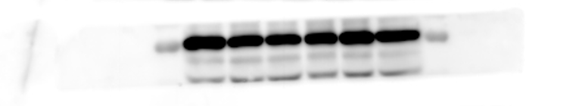

Supplement: Figure 1—source data 2. [file elife-99670-fig1-data2.zip › -Figure 1-source data 2. Original files for western blot analysis displayed in Figure 1A and 1D/Figure 1D/Tubulin.tif]

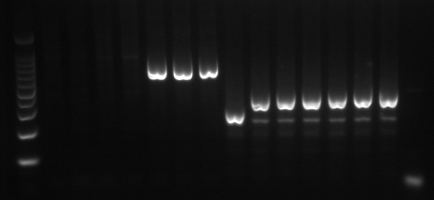

Supplement: Figure 1—figure supplement 1—source data 2. [file elife-99670-fig1-figsupp1-data2.zip › Figure 1–figure supplement 1-source data 2. Original files for western blot analysis displayed in Figure 1–figure supplement 1A and 1B/Figure 1–figure supplement 1A/AARS2-Cre.tif]

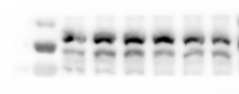

Supplement: Figure 1—figure supplement 1—source data 2. [file elife-99670-fig1-figsupp1-data2.zip › Figure 1–figure supplement 1-source data 2. Original files for western blot analysis displayed in Figure 1–figure supplement 1A and 1B/Figure 1–figure supplement 1B/AARS2-1.tif]

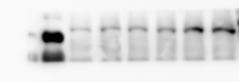

Supplement: Figure 1—figure supplement 1—source data 2. [file elife-99670-fig1-figsupp1-data2.zip › Figure 1–figure supplement 1-source data 2. Original files for western blot analysis displayed in Figure 1–figure supplement 1A and 1B/Figure 1–figure supplement 1B/AARS2-2.tif]

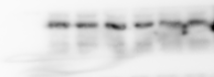

Supplement: Figure 1—figure supplement 1—source data 2. [file elife-99670-fig1-figsupp1-data2.zip › Figure 1–figure supplement 1-source data 2. Original files for western blot analysis displayed in Figure 1–figure supplement 1A and 1B/Figure 1–figure supplement 1B/AARS2-3.tif]

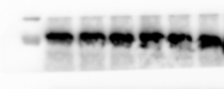

Supplement: Figure 1—figure supplement 1—source data 2. [file elife-99670-fig1-figsupp1-data2.zip › Figure 1–figure supplement 1-source data 2. Original files for western blot analysis displayed in Figure 1–figure supplement 1A and 1B/Figure 1–figure supplement 1B/Tubulin-1.tif]

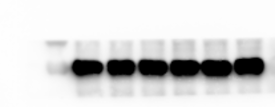

Supplement: Figure 1—figure supplement 1—source data 2. [file elife-99670-fig1-figsupp1-data2.zip › Figure 1–figure supplement 1-source data 2. Original files for western blot analysis displayed in Figure 1–figure supplement 1A and 1B/Figure 1–figure supplement 1B/Tubulin-2.tif]

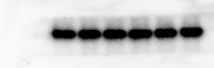

Supplement: Figure 1—figure supplement 1—source data 2. [file elife-99670-fig1-figsupp1-data2.zip › Figure 1–figure supplement 1-source data 2. Original files for western blot analysis displayed in Figure 1–figure supplement 1A and 1B/Figure 1–figure supplement 1B/Tubulin-3.tif]

Figure 2B

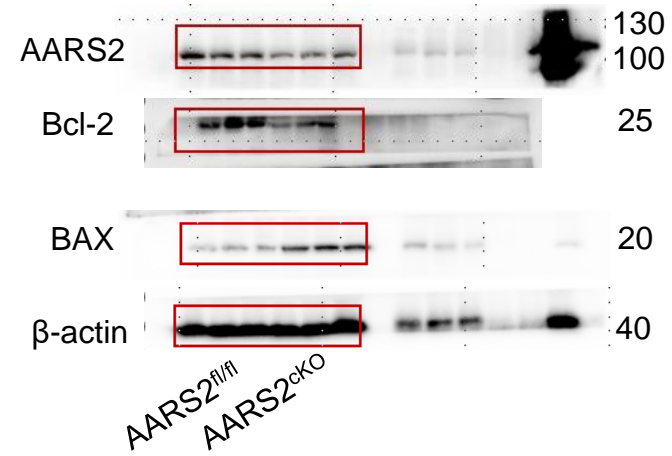

Supplement: Figure 2—source data 1. [file elife-99670-fig2-data1.zip › Figure 2-source data 1. PDF file containing original western blots for Figure 2B.pdf]

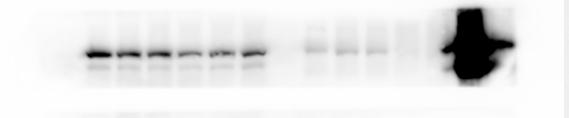

Supplement: Figure 2—source data 2. [file elife-99670-fig2-data2.zip › -Figure 2-source data 2. Original files for western blot analysis displayed in Figure 2B/Figure 2B/AARS2.tif]

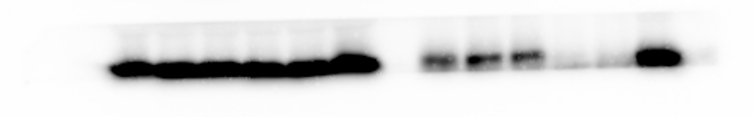

Supplement: Figure 2—source data 2. [file elife-99670-fig2-data2.zip › -Figure 2-source data 2. Original files for western blot analysis displayed in Figure 2B/Figure 2B/Actin.tif]

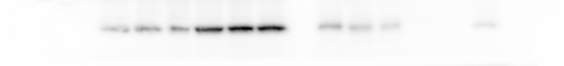

Supplement: Figure 2—source data 2. [file elife-99670-fig2-data2.zip › -Figure 2-source data 2. Original files for western blot analysis displayed in Figure 2B/Figure 2B/Bax.tif]

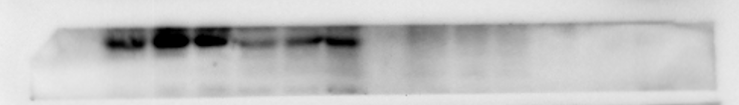

Supplement: Figure 2—source data 2. [file elife-99670-fig2-data2.zip › -Figure 2-source data 2. Original files for western blot analysis displayed in Figure 2B/Figure 2B/Bcl-2.tif]

Figure 2–figure  
supplement 1A

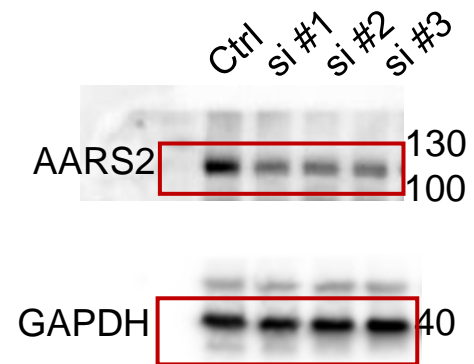

Figure 2–figure  
supplement 1B

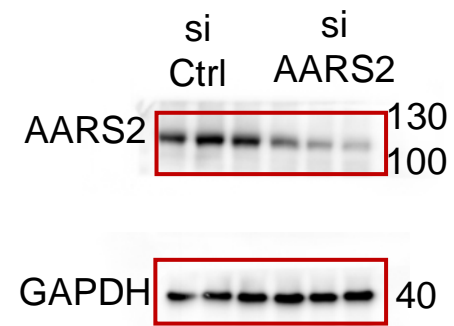

Supplement: Figure 2—figure supplement 1—source data 1. [file elife-99670-fig2-figsupp1-data1.zip › Figure 2–figure supplement 1-source data 1. PDF file containing original western blots for Figure 2–figure supplement 1A and 1B.pdf]

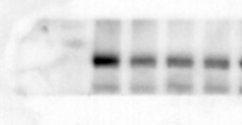

Supplement: Figure 2—figure supplement 1—source data 2. [file elife-99670-fig2-figsupp1-data2.zip › Figure 2–figure supplement 1-source data 2. Original files for western blot analysis displayed in Figure 2–figure supplement 1A and 1B/Figure 2–figure supplement 1A/AARS2.tif]

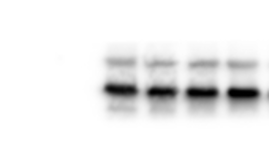

Supplement: Figure 2—figure supplement 1—source data 2. [file elife-99670-fig2-figsupp1-data2.zip › Figure 2–figure supplement 1-source data 2. Original files for western blot analysis displayed in Figure 2–figure supplement 1A and 1B/Figure 2–figure supplement 1A/GAPDH.tif]

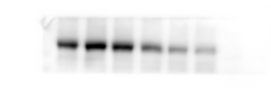

Supplement: Figure 2—figure supplement 1—source data 2. [file elife-99670-fig2-figsupp1-data2.zip › Figure 2–figure supplement 1-source data 2. Original files for western blot analysis displayed in Figure 2–figure supplement 1A and 1B/Figure 2–figure supplement 1B/AARS2.tif]

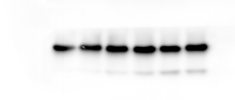

Supplement: Figure 2—figure supplement 1—source data 2. [file elife-99670-fig2-figsupp1-data2.zip › Figure 2–figure supplement 1-source data 2. Original files for western blot analysis displayed in Figure 2–figure supplement 1A and 1B/Figure 2–figure supplement 1B/GAPDH.tif]

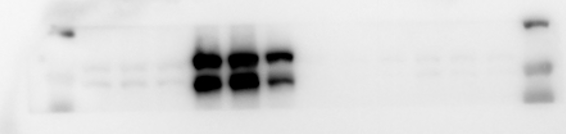

Supplement: Figure 3—source data 2. [file elife-99670-fig3-data2.zip › -Figure 3-source data 2. Original files for western blot analysis displayed in/Figure 3B/AARS2.tif]

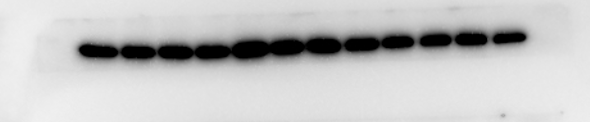

Supplement: Figure 3—source data 2. [file elife-99670-fig3-data2.zip › -Figure 3-source data 2. Original files for western blot analysis displayed in/Figure 3B/Actin.tif]

Figure 3—figure supplement 1A

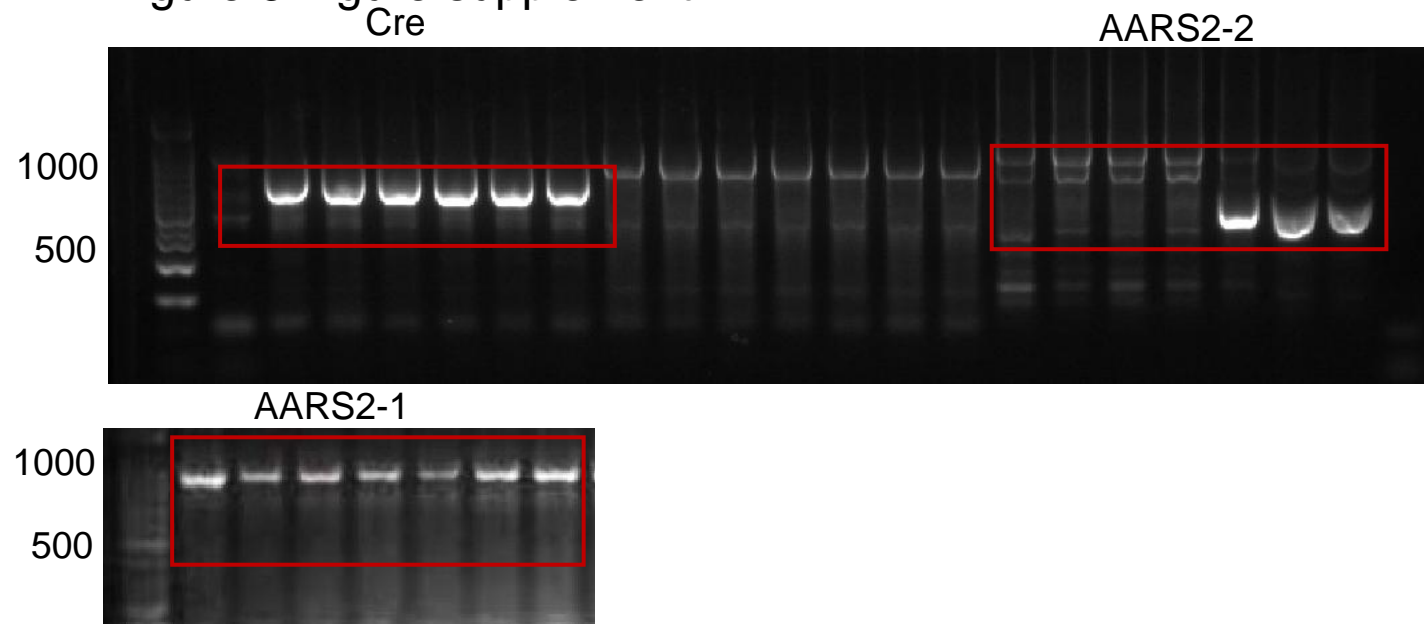

Figure 3—figure supplement 1B

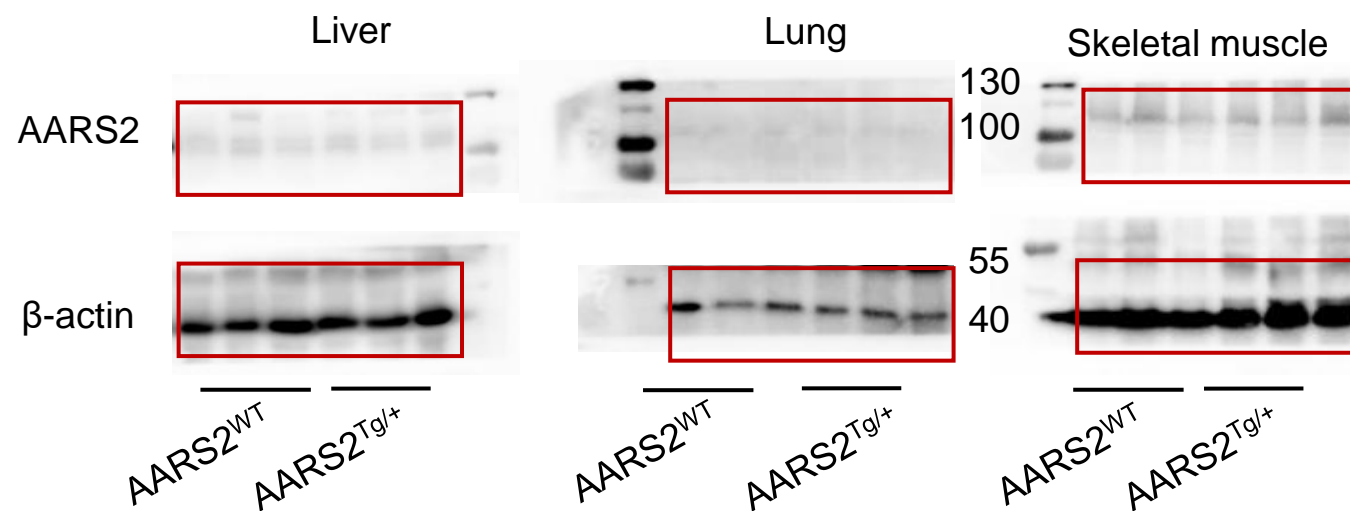

Supplement: Figure 3—figure supplement 1—source data 1. [file elife-99670-fig3-figsupp1-data1.zip › Figure 3–figure supplement 1-source data 1. PDF file containing original western blots for Figure 3–figure supplement 1A and 1B.pdf]

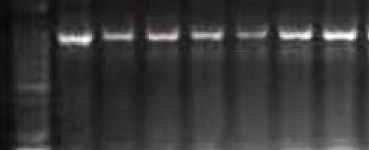

Supplement: Figure 3—figure supplement 1—source data 2. [file elife-99670-fig3-figsupp1-data2.zip › Figure 3–figure supplement 1-source data 2. Original files for western blot analysis displayed in/Figure 3–figure supplement 1A/AARS2-2.tif]

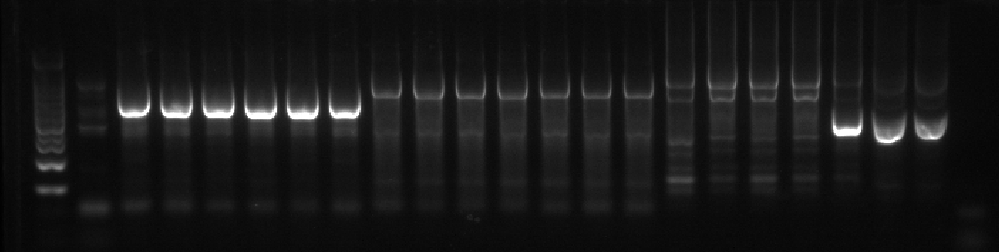

Supplement: Figure 3—figure supplement 1—source data 2. [file elife-99670-fig3-figsupp1-data2.zip › Figure 3–figure supplement 1-source data 2. Original files for western blot analysis displayed in/Figure 3–figure supplement 1A/AARS2-Cre.tif]

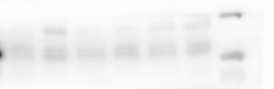

Supplement: Figure 3—figure supplement 1—source data 2. [file elife-99670-fig3-figsupp1-data2.zip › Figure 3–figure supplement 1-source data 2. Original files for western blot analysis displayed in/Figure 3–figure supplement 1B/AARS2-1.tif]

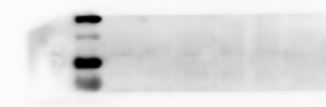

Supplement: Figure 3—figure supplement 1—source data 2. [file elife-99670-fig3-figsupp1-data2.zip › Figure 3–figure supplement 1-source data 2. Original files for western blot analysis displayed in/Figure 3–figure supplement 1B/AARS2-2.tif]

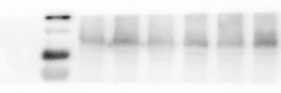

Supplement: Figure 3—figure supplement 1—source data 2. [file elife-99670-fig3-figsupp1-data2.zip › Figure 3–figure supplement 1-source data 2. Original files for western blot analysis displayed in/Figure 3–figure supplement 1B/AARS2-3.tif]

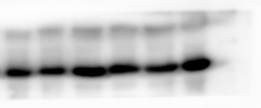

Supplement: Figure 3—figure supplement 1—source data 2. [file elife-99670-fig3-figsupp1-data2.zip › Figure 3–figure supplement 1-source data 2. Original files for western blot analysis displayed in/Figure 3–figure supplement 1B/actin-1.tif]

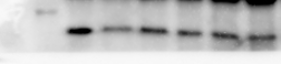

Supplement: Figure 3—figure supplement 1—source data 2. [file elife-99670-fig3-figsupp1-data2.zip › Figure 3–figure supplement 1-source data 2. Original files for western blot analysis displayed in/Figure 3–figure supplement 1B/actin-2.tif]

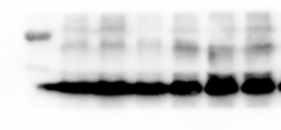

Supplement: Figure 3—figure supplement 1—source data 2. [file elife-99670-fig3-figsupp1-data2.zip › Figure 3–figure supplement 1-source data 2. Original files for western blot analysis displayed in/Figure 3–figure supplement 1B/actin-3.tif]

Figure 4B

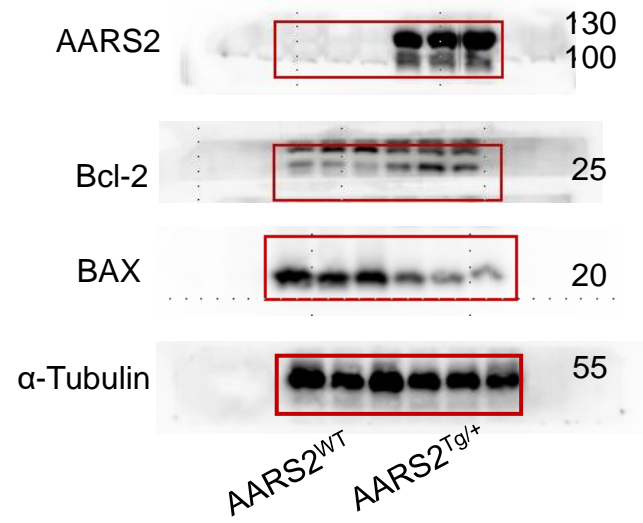

Figure 4E

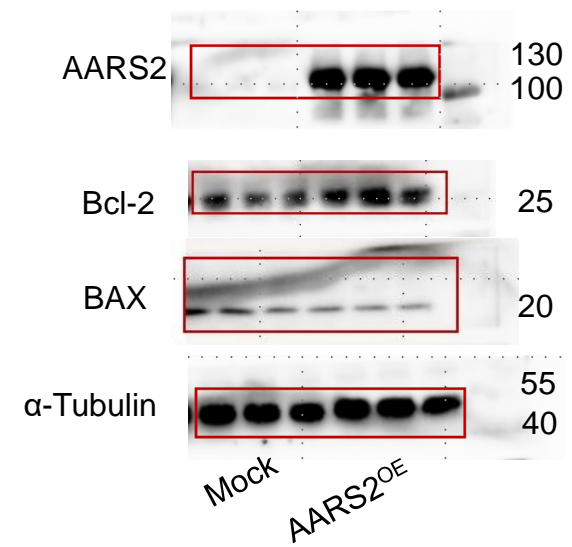

Supplement: Figure 4—source data 1. [file elife-99670-fig4-data1.zip › Figure 4-source data 1.PDF file containing original western blots for Figure 4B and 4E.pdf]

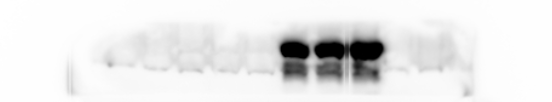

Supplement: Figure 4—source data 2. [file elife-99670-fig4-data2.zip › Figure 4-source data 2. Original files for western blot analysis displayed in Figure 4B and 4E/Figure 4B/AARS2.tif]

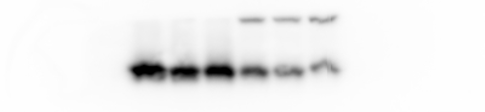

Supplement: Figure 4—source data 2. [file elife-99670-fig4-data2.zip › Figure 4-source data 2. Original files for western blot analysis displayed in Figure 4B and 4E/Figure 4B/BAX.png]

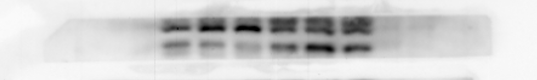

Supplement: Figure 4—source data 2. [file elife-99670-fig4-data2.zip › Figure 4-source data 2. Original files for western blot analysis displayed in Figure 4B and 4E/Figure 4B/Bcl-2.tif]

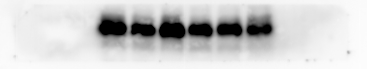

Supplement: Figure 4—source data 2. [file elife-99670-fig4-data2.zip › Figure 4-source data 2. Original files for western blot analysis displayed in Figure 4B and 4E/Figure 4B/Tubulin.tif]

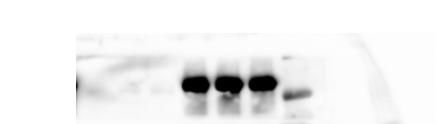

Supplement: Figure 4—source data 2. [file elife-99670-fig4-data2.zip › Figure 4-source data 2. Original files for western blot analysis displayed in Figure 4B and 4E/Figure 4E/AARS2.tif]

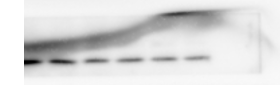

Supplement: Figure 4—source data 2. [file elife-99670-fig4-data2.zip › Figure 4-source data 2. Original files for western blot analysis displayed in Figure 4B and 4E/Figure 4E/BAX.tif]

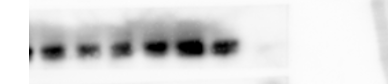

Supplement: Figure 4—source data 2. [file elife-99670-fig4-data2.zip › Figure 4-source data 2. Original files for western blot analysis displayed in Figure 4B and 4E/Figure 4E/BCL-2 (2).tif]

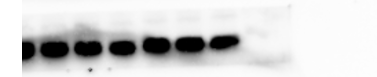

Supplement: Figure 4—source data 2. [file elife-99670-fig4-data2.zip › Figure 4-source data 2. Original files for western blot analysis displayed in Figure 4B and 4E/Figure 4E/Tubulin (2).tif]

Figure 6B

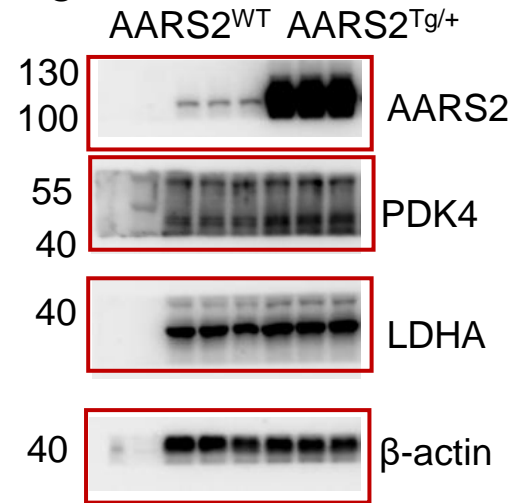

Figure 6C

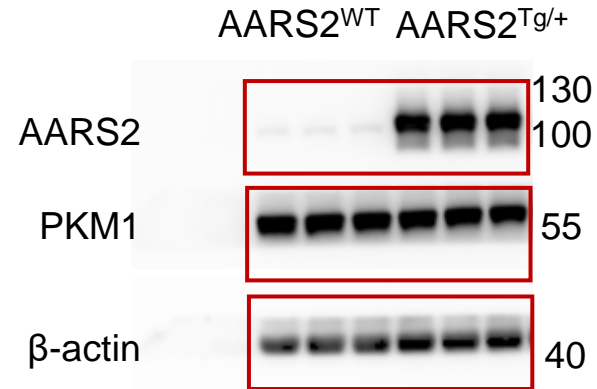

Figure 6D

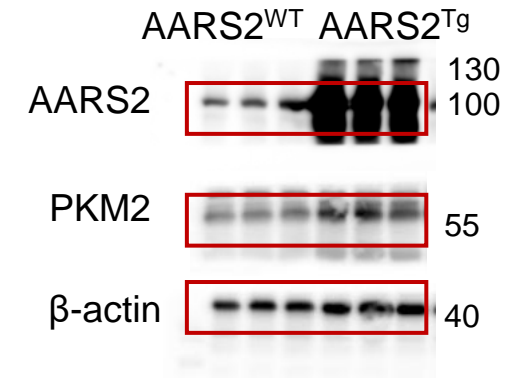

Figure 6E

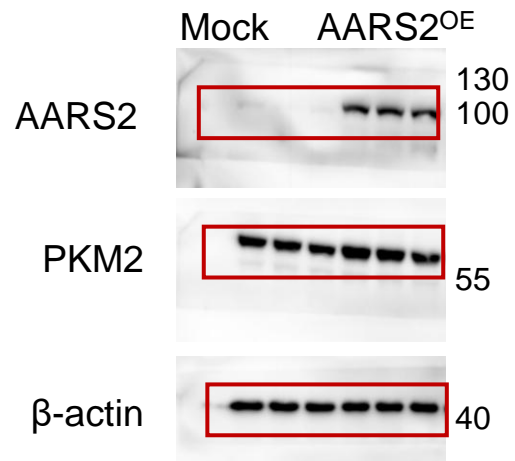

Figure 6F

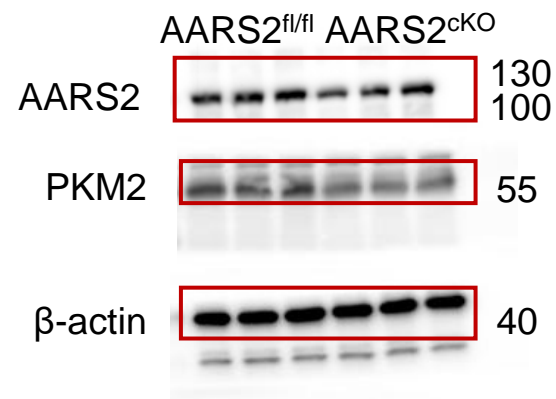

Figure 6G

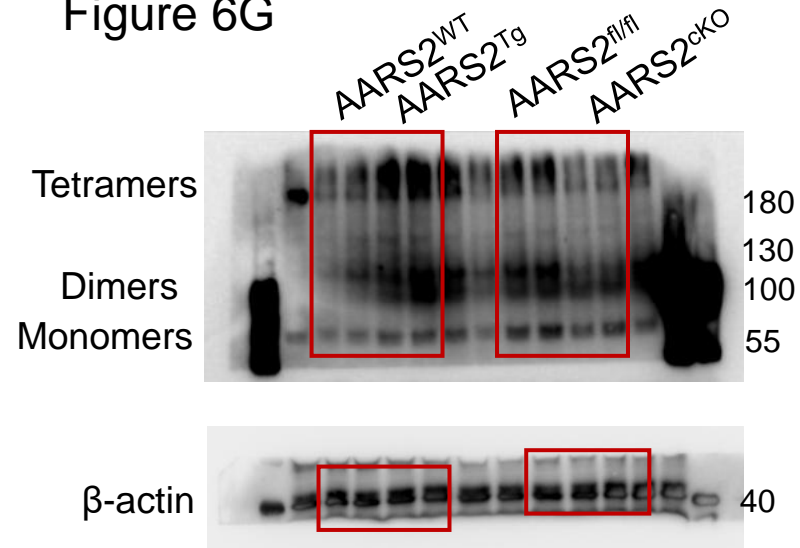

Figure 6K

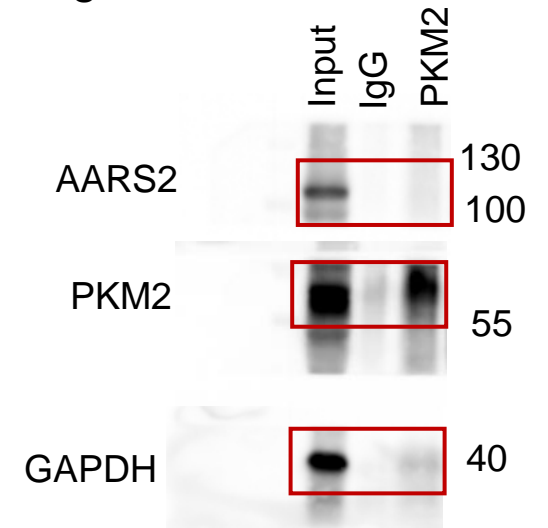

Supplement: Figure 6—source data 1. [file elife-99670-fig6-data1.zip › Figure 6-source data 1.PDF file containing original western blots for Figure 6B-6G and 6K.pdf]

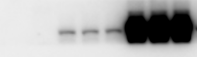

Supplement: Figure 6—source data 2. [file elife-99670-fig6-data2.zip › Figure 6-source data 2. Original files for western blot analysis displayed in Figure 6B-6G and 6K/Figure 6B/AARS2.tif]

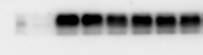

Supplement: Figure 6—source data 2. [file elife-99670-fig6-data2.zip › Figure 6-source data 2. Original files for western blot analysis displayed in Figure 6B-6G and 6K/Figure 6B/actin.tif]

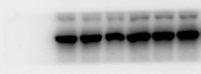

Supplement: Figure 6—source data 2. [file elife-99670-fig6-data2.zip › Figure 6-source data 2. Original files for western blot analysis displayed in Figure 6B-6G and 6K/Figure 6B/LDHA.tif]

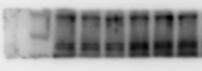

Supplement: Figure 6—source data 2. [file elife-99670-fig6-data2.zip › Figure 6-source data 2. Original files for western blot analysis displayed in Figure 6B-6G and 6K/Figure 6B/PDK4.tif]

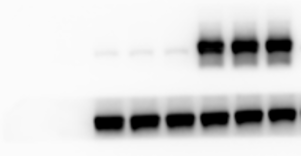

Supplement: Figure 6—source data 2. [file elife-99670-fig6-data2.zip › Figure 6-source data 2. Original files for western blot analysis displayed in Figure 6B-6G and 6K/Figure 6C/AARS2-PKM1.tif]

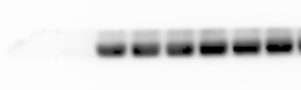

Supplement: Figure 6—source data 2. [file elife-99670-fig6-data2.zip › Figure 6-source data 2. Original files for western blot analysis displayed in Figure 6B-6G and 6K/Figure 6C/actin.tif]

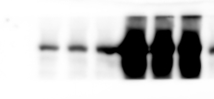

Supplement: Figure 6—source data 2. [file elife-99670-fig6-data2.zip › Figure 6-source data 2. Original files for western blot analysis displayed in Figure 6B-6G and 6K/Figure 6D/AARS2.tif]

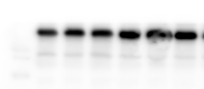

Supplement: Figure 6—source data 2. [file elife-99670-fig6-data2.zip › Figure 6-source data 2. Original files for western blot analysis displayed in Figure 6B-6G and 6K/Figure 6D/actin.tif]

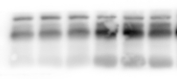

Supplement: Figure 6—source data 2. [file elife-99670-fig6-data2.zip › Figure 6-source data 2. Original files for western blot analysis displayed in Figure 6B-6G and 6K/Figure 6D/PKM2.tif]

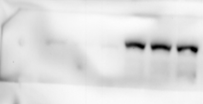

Supplement: Figure 6—source data 2. [file elife-99670-fig6-data2.zip › Figure 6-source data 2. Original files for western blot analysis displayed in Figure 6B-6G and 6K/Figure 6E/AARS2.tif]

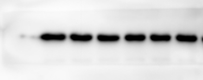

Supplement: Figure 6—source data 2. [file elife-99670-fig6-data2.zip › Figure 6-source data 2. Original files for western blot analysis displayed in Figure 6B-6G and 6K/Figure 6E/actin.tif]

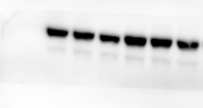

Supplement: Figure 6—source data 2. [file elife-99670-fig6-data2.zip › Figure 6-source data 2. Original files for western blot analysis displayed in Figure 6B-6G and 6K/Figure 6E/PKM2.tif]

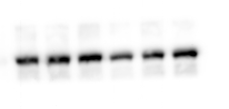

Supplement: Figure 6—source data 2. [file elife-99670-fig6-data2.zip › Figure 6-source data 2. Original files for western blot analysis displayed in Figure 6B-6G and 6K/Figure 6F/AARS2.tif]

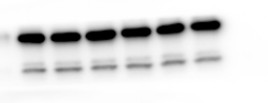

Supplement: Figure 6—source data 2. [file elife-99670-fig6-data2.zip › Figure 6-source data 2. Original files for western blot analysis displayed in Figure 6B-6G and 6K/Figure 6F/actin.tif]

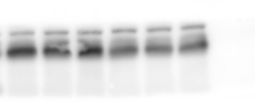

Supplement: Figure 6—source data 2. [file elife-99670-fig6-data2.zip › Figure 6-source data 2. Original files for western blot analysis displayed in Figure 6B-6G and 6K/Figure 6F/PKM2.tif]

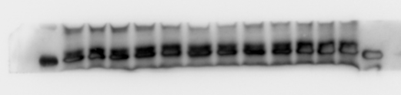

Supplement: Figure 6—source data 2. [file elife-99670-fig6-data2.zip › Figure 6-source data 2. Original files for western blot analysis displayed in Figure 6B-6G and 6K/Figure 6G/actin.tif]

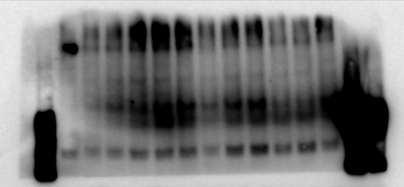

Supplement: Figure 6—source data 2. [file elife-99670-fig6-data2.zip › Figure 6-source data 2. Original files for western blot analysis displayed in Figure 6B-6G and 6K/Figure 6G/PKM2.tif]

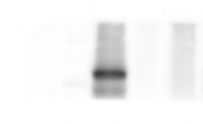

Supplement: Figure 6—source data 2. [file elife-99670-fig6-data2.zip › Figure 6-source data 2. Original files for western blot analysis displayed in Figure 6B-6G and 6K/Figure 6K/AARS2.tif]

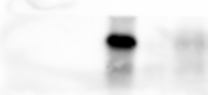

Supplement: Figure 6—source data 2. [file elife-99670-fig6-data2.zip › Figure 6-source data 2. Original files for western blot analysis displayed in Figure 6B-6G and 6K/Figure 6K/GAPDH.tif]

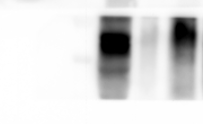

Supplement: Figure 6—source data 2. [file elife-99670-fig6-data2.zip › Figure 6-source data 2. Original files for western blot analysis displayed in Figure 6B-6G and 6K/Figure 6K/PKM2.tif]
