# Supplementary material for: AARS2 ameliorates myocardial ischemia via fine-tuning PKM2-mediated metabolism
Source: eLife. 2025 May 15;13:RP99670. doi: 10.7554/eLife.99670 (PMC12080999; doi:10.7554/eLife.99670)

Figure 3B

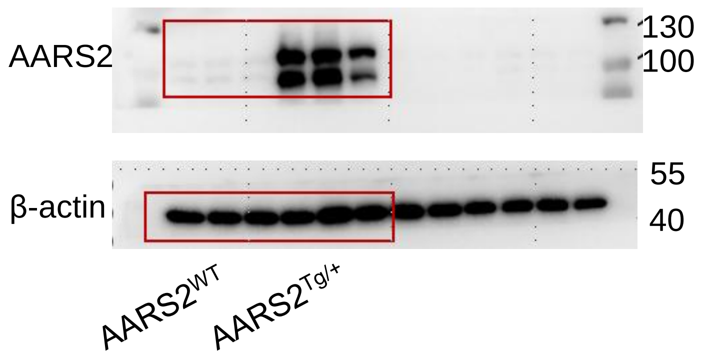

Supplement: Figure 3—source data 1. [file elife-99670-fig3-data1.zip › Figure 3-source data 1. PDF file containing original western blots for Figure 3B.pdf]
