# Supplementary material for: AARS2 ameliorates myocardial ischemia via fine-tuning PKM2-mediated metabolism
Source: eLife. 2025 May 15;13:RP99670. doi: 10.7554/eLife.99670 (PMC12080999; doi:10.7554/eLife.99670)
Supplement: Supplementary file 1. [file elife-99670-supp1.docx]

**Supplementary Data**

**A. *Aars2* siRNA** **sequences**

| Genes |  | Primers | Sequence (5' to 3') |
| --- | --- | --- | --- |
| *Aars2*-si-#1 |  | Forward | GCUUCCGACGAGUAGCUAACATT |
|  |  | Reverse | UGUUAGCUACUCGUCGGAAGCTT |
| *Aars2*-si-#2 |  | Forward | CGUUCGGACUGCAAGAGAACUTT |
|  |  | Reverse | AGUUCUCUUGCAGUCCGAACGTT |
| *Aars2*-si-#3 |  | Forward | GGAGGAGCUGCACCGUCAAGGTT |
|  |  | Reverse | CCUUGACGGUGCAGCUCCUCCTT |

**B. Genotyping primer sequences**

*Aars2* transgenic mouse primers

| Primer | Sequences (5'-3') |
| --- | --- |
| P1 Forward | TCAGATTCTTTTATAGGGGACACA |
| P1 Reverse  P2 Forward | TAAAGGCCACTCAATGCTCACTAA  TGCCCTCAGTATAGCCCAAACC |
| P2 Reverse | GCAGCCAAGGAAAGGACGATGATT |

The molecular weight of the two pairs of primers was the same, generating a WT band: 994bp; and Mut (mutant) band: 496bp.

*Aars2* knockout mouse primers

| Primers | Sequences (5' to 3') |
| --- | --- |
| P1 Forward | AAGCAACAGGAGAAGAGGTGTTGG |
| P1 Reverse | TAACCATCTCAGCAGCCCAGCAT |

The pair of primers generating a WT band: 268bp, and MUT band: 372bp.

*Cre* primers

| Primers | Sequences (5' to 3') |
| --- | --- |
| P1 Forward | AATGCTTCTGTCCGTTTGC |
| P1 Reverse | ACCAGAGTCATCCTTAGCG |

*Cre* primer pair generating a 712-bp band.

**C. Primers used for real-time PCR analysis**

| Genes | Primers | Sequences (5' to 3') |
| --- | --- | --- |
| rat *Pkm2* | Forward | GATCTGAAGTACGCCCGAGG |
|  | Reverse | GAATGAAGGCAGTCCCTGCT |
| rat *β-Actin* | Forward | AACCTTCTTGCAGCTCCTCC |
|  | Reverse | TACCCACCATCACACCCTGG |
| mouse *β-Actin* | Forward | GGCTGTATTCCCCTCCATCG |
|  | Reverse | CCAGTTGGTAACAATGCCATGT |
| mouse *Aars2* | Forward | AAGTTACGGTATGCTGAGCCG |
|  | Reverse | AACTACACGTCGGAAGCCTG |
| mouse *Pkm2* | Forward | TGTCTGGAGAAACAGCCAAG |
|  | Reverse | TCCTCGAATAGCTGCAAGTG |
